# Drug-Related Problems of Patients in Primary Health Care Institutions: A Systematic Review

**DOI:** 10.3389/fphar.2021.698907

**Published:** 2021-08-17

**Authors:** Xiao-Feng Ni, Chun-Song Yang, Yu-Mei Bai, Zi-Xian Hu, Ling-Li Zhang

**Affiliations:** ^1^ Department of Pharmacy, West China Second University Hospital, Sichuan University, Chengdu, China; ^2^ Evidence-Based Pharmacy Center, West China Second University Hospital, Sichuan University, Chengdu, China; ^3^ Key Laboratory of Birth Defects and Related Diseases of Women and Children, Ministry of Education (Sichuan University), Chengdu, China; ^4^ West China School of Medicine, Sichuan University, Chengdu, China; ^5^ West China School of Pharmacy, Sichuan University, Chengdu, China

**Keywords:** drug-related problems, primary health care, systematic review, medication review, pharmaceutical service

## Abstract

**Introduction:** Drug-related problems (DRPs) are not only detrimental to patients' physical health and quality of life but also lead to a serious waste of health care resources. The condition of DRPs might be more severe for patients in primary health care institutions.

**Objective**: This systematic review aims to comprehensively review the characteristics of DRPs for patients in primary health care institutions, which might help find effective strategies to identify, prevent, and intervene with DRPs in the future.

**Methods:** We searched three English databases (Embase, The Cochrane Library, and PubMed) and four Chinese databases (CNKI, CBM, VIP, and Wanfang). Two of the researchers independently conducted literature screening, quality evaluation, and data extraction. Qualitative and quantitative methods were combined to analyze the data.

**Results:** From the 3,368 articles screened, 27 met the inclusion criteria and were included in this review. The median (inter-quartile range, IQR) of the incidences of DRPs was 70.04% (59%), and the median (IQR) of the average number of DRPs per patient was 3.4 (2.8). The most common type of DRPs was “treatment safety.” The causes of DRPs were mainly in the prescribing section, including “drug selection” and “dose selection”, while patients' poor adherence in the use section was also an important cause of DRPs. Risk factors such as the number of medicines, age, and disease condition were positively associated with the occurrence of DRPs. In addition, the medians (IQR) of the rate of accepted interventions, implemented interventions, and solved DRPs were 78.8% (22.3%), 64.15% (16.85%), and 76.99% (26.09%), respectively.

**Conclusion:** This systematic review showed that the condition of DRPs in primary health care institutions was serious. In pharmaceutical practice, the patients with risk factors of DRPs should be monitored more closely. Pharmacists could play important roles in the identification and intervention of DRPs, and more effective intervention strategies need to be established in the future.

## Introduction

A drug-related problem (DRP) was defined by the Pharmaceutical Care Network Europe (PCNE) as an event or circumstance involving drug therapy that actually or potentially interferes with desired health outcomes (Pharmaceutical Care Network Europe Association, 2020), which mainly includes unnecessary drug treatment, inadequate drug treatment, ineffective drug treatment, adverse drug event, inappropriate dosage, and poor adherence (Strand et al., 1990; Frommer et al., 1992; Pharmaceutical Care Network Europe Association, 2020). Unresolved or potential DRPs could lead to unnecessary outpatient visits, hospital admissions, and long-term care, which not only interfered with clinical treatment but also increased patients' financial burden (Stafford et al., 2009; Ucha-Samartín et al., 2013; Lenssen et al., 2016). In the United States, DRPs were one of the main causes of death (Budnitz et al., 2006), with approximately 3–6% (700,000/ year) of hospital admissions and a cost of 130 billion dollars each year (World Health Organization, 2016 and International Pharmaceutical Federation, 2016). In Portugal, the number of hospital admissions due to DRPs was approximately 43,000/ year, which equated to about five patients per hour (Guerreiro et al., 2005). In brief, DRPs have caused damage to patients’ physical health and quality of life to a certain extent and have led to a significant waste of health care resources. Notably, up to 88% of DRPs could have been avoided (Beijer and de Blaey, 2002; Mcdonnell and Jacobs, 2002; Baena et al., 2006).

According to the World Health Organization (2021), primary health care is a whole-of-society approach to health that aims at ensuring the highest possible level of health and well-being and their equitable distribution by focusing on people’s needs and as early as possible. However, medical care falls short of what should be provided for many conditions and in many countries (Roland and Olesen, 2016). For example, recent research studies (Li et al., 2017; Su et al., 2017; Li et al., 2020) suggested that the quality of diagnosis and treatment was low in Chinese primary health care institutions, with problems such as overuse of antibiotics, inadequate treatment of noncommunicable diseases, and poor management of chronic diseases. In Haiti, despite an extensive network of health facilities, a minority of Haitians had access to a primary care facility of good quality, especially in rural areas (Gage et al., 2017). The factors such as poor governance, population growth, inadequate health systems, and scarce research and assessment on primary health care limited the development of primary health care (Walley et al., 2008). Therefore, more attention should be paid to the condition of DRPs in primary health care institutions; however, there is a lack of systematic review to comprehensively analyze the characteristics of DRPs for patients in primary health care institutions.

Therefore, this systematic review aimed to review the current studies related to DRPs of patients in primary health care institutions and gain insight into the characteristics of DRPs, including incidence, types, causes, risk factors, and the acceptability of interventions, which might be helpful to find effective strategies to identify, prevent, and intervene with DRPs in the future and improve the quality of primary health care services.

## Methods

This systematic review was conducted according to the Cochrane Handbook for Systematic Reviews of Interventions (version 5.1.0) (Higgins et al., 2011), and this systematic review was based on the Preferred Reporting Items for Systematic Reviews and Meta-analyses (PRISMA) statement (Liberati et al., 2009). All authors discussed the protocol many times based on the purpose of this systematic review and formulated the final protocol. The protocol is available on INPLASY with registration number INPLASY202160081.

### Search Strategy

Computer retrieval was conducted in three English databases (Embase, The Cochrane Library, and PubMed) and four Chinese databases (CNKI, CBM, VIP, and Wanfang), from their inception dates to December 17, 2020. Additionally, we manually searched Google, Baidu, and the reference lists of the included studies. We consulted an informatics expert and developed the search strategies that combined subject headings and free text terms (the search strategies are reported in Supplementary Appendix S1).

### Inclusion and Exclusion Criteria

Inclusion criteria were as follows: 1) patients visiting primary health care institutions, including community health service centers, community health service stations, street health centers, township health centers, village health offices, and outpatient departments and clinics (infirmaries) (National Health And Family Planning Commission, 2020); 2) relevant studies reporting the characteristics of DRPs, including the incidence, types, causes, risk factors, and the acceptability of interventions.

Exclusion criteria were as follows: 1) duplicate publications; 2) articles not in English or Chinese; 3) articles without an available full text. The types of study design were not limited as this research aimed to conduct a comprehensive review of all published studies.

### Study Selection

According to the predefined criteria, two researchers (XF Ni and YM Bai) independently screened the titles and abstracts of articles. Then, the screenings of full texts were conducted by the 2 researchers for potential eligible articles after preliminary screening. After cross-checking, disagreements were resolved by discussions between the two researchers and the remaining ones were decided by a third researcher (CS Yang). For intervention studies, we only focused on the study phase before the interventions.

### Quality Assessment

Currently, there were no more accepted quality assessment instruments for cross-sectional studies (Sanderson et al., 2007). After discussions, we decided to choose a relatively widely used scale, the Agency for Healthcare Research and Quality (AHRQ) scale. Because we only focused on the pre-intervention phase, the quality of intervention studies was also evaluated using the Agency for Healthcare Research and Quality scale. Two researchers (XF Ni and YM Bai) independently evaluated the quality of the included articles using the AHRQ scale with 11 items, each of which was answered with “yes,” “no,” and “unclear.” Any disagreements after cross-checking were resolved by discussions between the two researchers, with the final decision being made by the third researcher (CS Yang). If the answer was “no,” “unclear,” or “not applicable,” the item was given a score of “0”; if the answer was “yes,” the item was scored as “1.” The quality assessments of the articles were classified as follows: low quality = 0–3; medium quality = 4–7; high quality = 8–11 (Hu et al., 2015; Zeng et al., 2015).

### Data Extraction

Two researchers (XF Ni and YM Bai) independently extracted data according to the data collection form designed in advance. The two researchers discussed the disagreements and resolved them with the help of the third researcher (CS Yang). For articles with incomplete information, corresponding authors were contacted whenever possible to obtain detailed information. For intervention studies, we only extracted the characteristics of DRPs before the interventions.

### Statistical Analysis

A combination of qualitative and quantitative analysis was used in this research. If the initial data were not expressed as the mean and standard deviation, the sample size, median, range, and/or inter-quartile range (IQR) were used to estimate the mean and standard deviation for comparison purposes (Wan et al., 2014; Luo et al., 2018). PCNE Classification for Drug-Related Problems is used for researching the nature, prevalence, and incidence of DRPs, which contains primary domains and subdomains for types and causes. Those subdomains can be seen as explanatory for the primary domains. As different included studies chose different DPRs classification systems, we reclassified the types and causes of DPRs according to the PCNE Classification for Drug-Related Problems Version 9.1 (Plácido et al., 2020); DRPs were only classified in the primary domain when there was not enough information to make a clear classification in a particular subdomain.

## Results

### Study Selection

**FIGURE 1:**
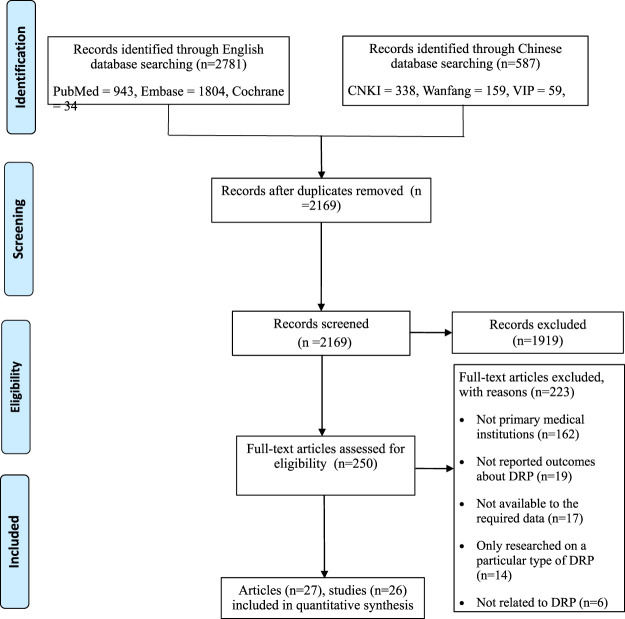
The process of study selection.

The systematic search yielded a total of 2,781 English and 587 Chinese records. After preliminary screening, 250 articles needed to be further screened. Twenty-seven articles (Patel et al., 2005; Gisev et al., 2009; Gomez et al., 2009; Hooper et al., 2009; Zhao, 2012; Roth et al., 2013; Lenander et al., 2014; Nadir et al., 2014; Tan et al., 2014; Mendonca et al., 2016; Okumura et al., 2016; Rodis et al., 2017; Schwartz et al., 2017; Benson et al., 2018; Vande Griend et al., 2018; Yang et al., 2018; Hazen et al., 2019; Khera et al., 2019; Neves et al., 2019; Santos et al., 2019; Zhang et al., 2020a; Zhang et al., 2020b; Chung et al., 2020; Gerard et al., 2020; Puspitasari et al., 2020; Samir Abdin et al., 2020; Troncoso-Marino et al., 2020) were finally included (there were only 26 studies because one of the studies was reported in two articles) (Zhang et al., 2020a; Zhang et al., 2020b). The process of study selection is shown in Figure 1.

### Quality Assessment

All the included studies were assessed by the AHRQ scale. One study was of high quality (Zhao, 2012), 25 studies were of medium quality, and none were of low quality. The quality problems were mainly due to the following items: Q5 = if evaluators of subjective components of the study were masked to other aspects of the status of the participants (*n* = 26, 100%); Q9 = if applicable, it explains how missing data were handled in the analysis (*n* = 24, 92.3%); Q4 = whether or not subjects were consecutive if not population-based (*n* = 21, 80.8%); Q7 = any patient exclusions from analysis (*n* = 18, 69.2%); Q6 = any assessments undertaken for quality assurance purposes (*n* = 15, 57.7%). The results of quality assessment for each study were shown in Table 1.

**TABLE 1:** Quality assessment of included studies.

Study	Q1	Q2	Q3	Q4	Q5	Q6	Q7	Q8	Q9	Q10	Q11	Score
Benson et al.	Y	Y	Y	U	U	Y	U	Y	U	Y	NA	6
Chung et al.	Y	Y	Y	U	U	Y	U	N	U	Y	NA	5
Gerard et al.	Y	Y	N	Y	U	N	U	N	U	Y	NA	4
Gisev et al.	Y	Y	N	U	U	Y	U	Y	U	Y	Y	6
Gomez et al.	Y	Y	Y	N	U	N	Y	Y	U	Y	NA	6
Hazen et al.	Y	Y	Y	Y	U	Y	U	N	U	Y	NA	6
Hooper et al.	Y	Y	Y	U	U	Y	U	N	U	Y	NA	4
Khera et al.	Y	Y	Y	U	U	N	U	Y	U	Y	NA	5
Lenander et al.	Y	Y	Y	U	U	Y	U	N	U	Y	Y	6
Mendonca et al.	Y	Y	Y	Y	U	N	U	Y	U	Y	Y	7
Nadir et al.	Y	Y	Y	U	U	Y	U	Y	U	Y	NA	6
Neves et al.	Y	N	Y	U	U	Y	U	Y	U	Y	NA	5
Okumura et al.	Y	Y	Y	U	U	N	Y	N	U	Y	NA	5
Patel et al.	Y	Y	Y	N	U	N	U	Y	U	Y	NA	5
Puspitasari et al.	Y	Y	Y	Y	U	N	Y	Y	U	Y	NA	7
Rodis et al.	Y	Y	Y	U	U	N	U	N	Y	Y	NA	5
Roth et al.	Y	Y	Y	N	U	N	Y	Y	U	Y	Y	7
Samir Abdin et al.	Y	Y	Y	U	U	N	U	N	U	Y	Y	5
Santos et al.	Y	Y	Y	Y	U	N	U	Y	U	Y	N	6
Schwartz et al.	Y	Y	Y	N	U	N	Y	N	U	Y	NA	5
Tan et al.	Y	Y	Y	U	U	Y	U	N	U	Y	Y	6
Troncoso-Marino et al.	Y	Y	Y	U	U	N	U	Y	U	Y	NA	5
Vande Griend et al.	Y	N	Y	U	U	Y	U	Y	U	Y	NA	5
Yang et al.	Y	Y	Y	U	U	N	Y	Y	U	Y	Y	7
Zhang et al.	Y	Y	Y	N	U	N	Y	Y	U	Y	NA	6
Zhao	Y	Y	Y	N	U	Y	Y	Y	Y	Y	NA	8

Note: Y = yes; N = no; U = unclear; NA = not available.

Q1: the source of information (survey, record review); Q2: listing inclusion and exclusion criteria for exposed and unexposed subjects (cases and controls) or referring to previous publications; Q3: time period used for identifying patients; Q4: whether or not subjects were consecutive if not population-based; Q5: if evaluators of subjective components of the study were masked to other aspects of the status of the participants; Q6: any assessments undertaken for quality assurance purposes (e.g., test/retest of primary outcome measurements); Q7: any patient exclusions from analysis; Q8: how confounding was assessed and/or controlled; Q9: if applicable, it explains how missing data were handled in the analysis; Q10: summarizing patient response rates and completeness of data collection; Q11: clarifying what follow-up, if any, was expected and the percentage of patients for which incomplete data or follow-up was obtained.

### Study Characteristics

Among the included studies, 61.54% (*n* = 16) were from developed countries, including six from America (Patel et al., 2005; Roth et al., 2013; Rodis et al., 2017; Schwartz et al., 2017; Vande Griend et al., 2018; Chung et al., 2020), three from Canada (Khera et al., 2019; Gerard et al., 2020; Samir Abdin et al., 2020), three from Australia (Gisev et al., 2009; Tan et al., 2014; Benson et al., 2018), two from Spain (Gomez et al., 2009; Troncoso-Marino et al., 2020), one from the Netherlands (Hazen et al., 2019), and one from Sweden (Lenander et al., 2014); 38.46% (*n* = 10) were from developing countries, including four from Brazil (Mendonca et al., 2016; Okumura et al., 2016; Neves et al., 2019; Santos et al., 2019), three from China (Zhao, 2012; Yang et al., 2018; Zhang et al., 2020a; Zhang et al., 2020b), two from Qatar (Hooper et al., 2009; Nadir et al., 2014), and one from Indonesia (Puspitasari et al., 2020). According to the types of study design reported by the authors, there were six prospective studies (Hooper et al., 2009; Roth et al., 2013; Rodis et al., 2017; Benson et al., 2018; Yang et al., 2018; Zhang et al., 2020a; Zhang et al., 2020b), five cross-sectional studies (Zhao, 2012; Nadir et al., 2014; Okumura et al., 2016; Hazen et al., 2019; Troncoso-Marino et al., 2020), four retrospective studies (Mendonca et al., 2016; Vande Griend et al., 2018; Chung et al., 2020; Gerard et al., 2020), five before-and-after controlled or longitudinal studies (Gomez et al., 2009; Tan et al., 2014; Khera et al., 2019; Neves et al., 2019; Samir Abdin et al., 2020), and one randomized controlled study (Lenander et al., 2014). In addition, one study included both cross-sectional and retrospective study phases (Puspitasari et al., 2020), one study included both cross-sectional and quasi-experimental study phases (Santos et al., 2019), and the remaining three studies did not report the types of study design (Patel et al., 2005; Gisev et al., 2009; Schwartz et al., 2017). The number of research centers ranged from 1 to 284, the sample size from 48 to 916,619, and the study duration from 1 to 29 months. The study subjects of 11 studies were older adults (Zhao, 2012; Roth et al., 2013; Lenander et al., 2014; Mendonca et al., 2016; Schwartz et al., 2017; Yang et al., 2018; Hazen et al., 2019; Khera et al., 2019; Zhang et al., 2020a; Zhang et al., 2020b; Gerard et al., 2020; Troncoso-Marino et al., 2020), seven studies with adults (Patel et al., 2005; Gisev et al., 2009; Nadir et al., 2014; Rodis et al., 2017; Santos et al., 2019; Chung et al., 2020; Samir Abdin et al., 2020), and two studies with children (Okumura et al., 2016; Puspitasari et al., 2020); six studies did not have strict restrictions on the age of study subjects (Gomez et al., 2009; Hooper et al., 2009; Tan et al., 2014; Benson et al., 2018; Vande Griend et al., 2018; Neves et al., 2019). One study focused on acute diseases (Puspitasari et al., 2020), six studies focused on chronic diseases (Patel et al., 2005; Gisev et al., 2009; Zhao, 2012; Rodis et al., 2017; Yang et al., 2018; Zhang et al., 2020a; Zhang et al., 2020b), and 13 studies focused on diseases with complex treatment needs such as polypharmacy and multiple comorbidities (Gomez et al., 2009; Roth et al., 2013; Lenander et al., 2014; Nadir et al., 2014; Tan et al., 2014; Mendonca et al., 2016; Schwartz et al., 2017; Benson et al., 2018; Hazen et al., 2019; Khera et al., 2019; Chung et al., 2020; Gerard et al., 2020; Samir Abdin et al., 2020); six studies did not have strict restrictions on the disease type of study subjects (Hooper et al., 2009; Okumura et al., 2016; Vande Griend et al., 2018; Neves et al., 2019; Santos et al., 2019; Troncoso-Marino et al., 2020). Nine studies reported the average number of comorbidities ranged from 2.3 to 8.5 (Gisev et al., 2009; Zhao, 2012; Roth et al., 2013; Lenander et al., 2014; Mendonca et al., 2016; Benson et al., 2018; Yang et al., 2018; Santos et al., 2019; Zhang et al., 2020a; Zhang et al., 2020b), and 15 studies reported the average number of medicines used at the same time ranged from 4.2 to 14.2 (Patel et al., 2005; Gisev et al., 2009; Gomez et al., 2009; Zhao, 2012; Roth et al., 2013; Lenander et al., 2014; Tan et al., 2014; Mendonca et al., 2016; Schwartz et al., 2017; Benson et al., 2018; Vande Griend et al., 2018; Neves et al., 2019; Santos et al., 2019; Zhang et al., 2020a; Zhang et al., 2020b; Samir Abdin et al., 2020). The information of study characteristics is detailed in Table 2.

**TABLE 2:** The information of the study characteristics and study outcomes.

First author, year, country	Study design^a^	Number of research centers	Male/Sample size	Study duration (months)	Age group^b^	Disease type	Number of diseases (mean ± SD; range)	Number of medications (mean ± SD; Range)	Medicine review		
									Scope^c^	Interviews	DRP classification system
Chung et al., 2020, America	Retrospective observational study	1	1,258/3,280	12	2, 3	Chronic disease and polypharmacy therapy	1: 51.95%; 2: 39.12%; 3: 7.71%; 4: 1.16%; 5: 0.06%	≥4: 100%	NR	N	Pharmacotherapy workup notes
Vande Griend et al. (2018), America	Retrospective cohort study	1	121/280	9	4	No limit	NR	5.5	NR	N	NR
Rodis et al., 2017, America	Prospective study	3	NR/706	22	2, 3	Chronic disease	1: 78.75%; 2: 21.25%	NR	NR	Y	NR
Schwartz et al., 2017, America	NR	2	27/50	3	3	Polypharmacy therapy	NR	12.1 ± 4.6	NR	Y	NR
Patel et al., 2005, America	NR	7	40/119	7	2, 3	Chronic disease	1: 66%; 2: 34%	6.4 ± 2.9	5	N	Strand et al.
Roth et al., 2013, America	Prospective observational study	1	27/64	6	3	Polypharmacy therapy	8.5 (3–14)	13.9 (5–31)	1, 2, 3	Y	MRP classification tool
Zhang et al., 2020a; Zhang et al., 2020b, China	Prospective study	2	193/412	7	3	Chronic disease	5.72 ± 2.2	6.7 ± 3.41	NR	Y	PCNE 8.03
Yang et al., 2018, China	Prospective study	1	36/102	15	3	Chronic disease	3.2	Chronic medications: 6.3 ± 3.2; traditional Chinese patent medicines: 1.4 ± 1.4; dietary supplements: 0.4 ± 0.8	1, 2, 4	Y	DOCUMENT
Zhao, 2012, China	Cross-sectional study	3	209/416	3	3	Chronic disease	3.5 ± 1.1	4.9	1, 2, 4, 5	Y	NR
Gerard et al., 2020, Canade	Retrospective observational study	13	107/237	1	3	Polypharmacy therapy	NR	Prescriptions: 9.2 ± 4.7; non-prescriptions: 2.1 ± 2.3; natural or herbal products: 0.4 ± 0.9; other medications: 0.1 ± 0.8	1, 2, 3, 5	Y	NR
Samir Abdin et al. (2020), Canade	Real-life before-and-after intervention study	4	42/60	3.5	2, 3	Polypharmacy therapy and multiple chronic diseases	NR	14.2 (0–15)	NR	Y	Strand et al.
Khera et al., 2019, Canade	Before-and-afterintervention study	1	21/54	14	3	Polypharmacy therapy or multiple chronic diseases	NR	<10: 33.3%; ≥10: 66.7%	1, 2	Y	NR
Troncoso-Marino et al., 2020, Spain	Cross-sectional study	284	387729/916619	12	3	No limit	1: 6.93%; 2–4: 29.33%; 5–9: 50.59%; ≥10: 13.15	0: 12.46%; 1: 6.21%; 2–4: 27.80%; 5–9: 38.98%; ≥10: 14.54%	5	N	NR
Gomez et al., 2009, Spain	Prospective longitudinal study	2	185/422	6	4	Polypharmacy therapy	NR	8.1 ± 2.4	NR	Y	Granada Ⅱ
Puspitasari et al., 2020, Indonesia	Phase I: Cross-sectional, phase II: retrospective study	1	99/179	6	1	Acute disease	NR	NR	NR	N	Pharmacotherapy workup notes
Santos et al., 2019, Brazil	Phase I: Cross-sectional study, phase II: quasi-experimental study	1	408/1,057	29	2, 3	No limit	2.3 ± 1.2 (0–8)	4.2 ± 1.2 (0–20)	1, 2, 4	Y	Pharmacotherapy workup notes
Neves et al., 2019, Brazil	Longitudinal study	2	35/90	24	4	No limit	0–2: 31.1%; ≥3: 68.9%	7.6 ± 2.7 (2–18)	1, 2, 4	N	Pharmacotherapy workup notes
Mendonca et al., 2016, Brazil	Retrospective study	3	30/92	28	3	Chronic disease and polypharmacy therapy	3.5	6	NR	N	Pharmacotherapy workup notes
Okumura et al., 2016, Brazil	Cross-sectional study	1	22/53	5	1	No limit	NR	NR	NR	N	NR
Hazen et al., 2019, Netherlands	Cross-sectional study	9	70/270	12	3	Chronic disease and polypharmacy therapy	Median (IQR): 6 (3)	Median (IQR): 8 (5)	NR	Y	Systematic tool to reduce inappropriate prescribing
Benson et al., 2018, Australia	Prospective observational study	15	NR/493	6	4	Polypharmacy therapy and/or multiple disease	5.5 ± 2.7	9.2 ± 4.3	1, 2	Y	Basger et al.
Gisev et al., 2009, Australia	NR	5	26/48	NR	2, 3	Chronic disease	3.5 ± 2.1	7.0 ± 4.6	NR	Y	Gilbert et al.
Nadir et al., 2014, Qatar	Cross-sectional study	1	41/52	3	2, 3	Chronic disease and polypharmacy therapy	3: 40%	≥5: ≥50%	NR	Y	PCNE 6.2
Hooper et al., 2009, Qatar	Prospective study	4	478/594	3	4	No limit	NR	NR	1	N	PCNE 5.01
Lenander et al., 2014, Sweden	Randomized controlled trial	1	45/141	15	3	Polypharmacy therapy	4.9 ± 1.92	8.0 ± 3.36	1, 2, 3	Y	Strand et al.
Tan et al., 2014, Australia	Prospective, before-and-after intervention study	2	32/82	6	4	Polypharmacy therapy, multiple chronic diseases, etc.	NR	11.5 ± 1.0	NR	Y	Strand et al.

NR = not reported; N = no; Y = yes.

a According to the authors' designation in the methods section.

b 1 = children; 2 = young or middle-aged people; 3 = old people; 4 = all ages.

c 1 = prescriptions; 2 = over-the-counter drugs; 3 = nature or herbal products; 4 = health supplements; 5 = others.

### Study Outcomes

DRPs were identified by pharmacists, pharmaceutical postgraduates, or researchers in medication reviews. Twelve studies reported the scope of medication reviews (Patel et al., 2005; Hooper et al., 2009; Zhao, 2012; Roth et al., 2013; Lenander et al., 2014; Benson et al., 2018; Yang et al., 2018; Khera et al., 2019; Neves et al., 2019; Santos et al., 2019; Gerard et al., 2020; Troncoso-Marino et al., 2020). In addition to prescription drugs, only nine studies reported medication reviews of over-the-counter drugs, natural or herbal medicines, and health supplements (Zhao, 2012; Roth et al., 2013; Lenander et al., 2014; Benson et al., 2018; Yang et al., 2018; Khera et al., 2019; Neves et al., 2019; Santos et al., 2019; Gerard et al., 2020), as their medication information was not usually listed in medical records. Another study conducted medication reviews only for systemic drugs (Troncoso-Marino et al., 2020). Seventeen studies reported that patients were involved in medication reviews through interviews (Gisev et al., 2009; Gomez et al., 2009; Zhao, 2012; Roth et al., 2013; Lenander et al., 2014; Nadir et al., 2014; Tan et al., 2014; Rodis et al., 2017; Schwartz et al., 2017; Benson et al., 2018; Yang et al., 2018; Hazen et al., 2019; Khera et al., 2019; Santos et al., 2019; Zhang et al., 2020a; Zhang et al., 2020b; Gerard et al., 2020; Samir Abdin et al., 2020). Only five studies reported the criteria used to identify DRPs in drug selection: one study used a structured implicit review (Chung et al., 2020), two studies used the Beers criteria (Zhao, 2012; Lenander et al., 2014), one study used the Beers, STOPP/START criteria, and implicit criteria (Khera et al., 2019), and one study used a self-made form to identify potential safety problems for drugs or drug interactions (Troncoso-Marino et al., 2020). A total of 18 studies reported DRPs classification systems: four studies used the classification system proposed by Strand et al. (Patel et al., 2005; Lenander et al., 2014; Tan et al., 2014; Samir Abdin et al., 2020), three studies used the PCNE classification systems (versions 8.03, 6.2, 5.01) (Hooper et al., 2009; Nadir et al., 2014; Zhang et al., 2020a; Zhang et al., 2020b), five studies used Pharmacotherapy Workup Notes proposed by Cipolle et al. (Mendonca et al., 2016; Neves et al., 2019; Santos et al., 2019; Chung et al., 2020; Puspitasari et al., 2020). Additionally, the Document classification system (Yang et al., 2018), the coding framework-based classification system proposed by Gilbert et al. (Gisev et al., 2009), the classification system proposed by Basger et al. (Benson et al., 2018) and the established criteria for the Spanish classification of DRPs (Granada Ⅱ) (Gomez et al., 2009), the Systematic Tool to Reduce Inappropriate Prescribing (Hazen et al., 2019), and the Medication-Related Problem Classification Tool (Roth et al., 2013) were used in one study, respectively. The information of study outcomes is detailed in Table 2.

### The Incidence and Classification of DRPs

The incidence of DRPs was reported in 14 studies and ranged from 8.54 to 99.16%, with a median (IQR) of 70.04% (59%) (Patel et al., 2005; Gomez et al., 2009; Zhao, 2012; Tan et al., 2014; Okumura et al., 2016; Benson et al., 2018; Vande Griend et al., 2018; Yang et al., 2018; Santos et al., 2019; Zhang et al., 2020a; Zhang et al., 2020b; Chung et al., 2020; Gerard et al., 2020; Samir Abdin et al., 2020; Troncoso-Marino et al., 2020). Sixteen studies reported that the average number of DRPs per patient ranged from 0.58 to 7.2, with a median (IQR) of 3.4 (2.8) (Patel et al., 2005; Gisev et al., 2009; Gomez et al., 2009; Zhao, 2012; Roth et al., 2013; Lenander et al., 2014; Nadir et al., 2014; Mendonca et al., 2016; Schwartz et al., 2017; Benson et al., 2018; Yang et al., 2018; Hazen et al., 2019; Neves et al., 2019; Zhang et al., 2020a; Zhang et al., 2020b; Gerard et al., 2020; Samir Abdin et al., 2020). The incidence of DRPs and the average number of DRPs per patient of included studies are detailed in Table 3.

**TABLE 3:** The incidence of DRPs and the mean number of DRPs per patient of included studies.

Study	The incidence of DRPs had at least one DRP^a^	The number of DRPs per Patient (mean ± SD; range)^b^
Benson et al.	93.91%	2.3 ± 1.3
Chung et al.	8.54%	—
Gerard et al.	98.73%	3.6 ± 2.8
Gisev et al.	—	4.4 ± 2.0
Gomez et al.	45.73%	0.58
Hazen et al.	—	4.8 ± 1.9 (1–12)
Hooper et al.	—	—
Khera et al.	—	—
Lenander et al.	—	1.6 ± 1.35
Mendonca et al.	—	3.4
Nadir et al.	—	3.4
Neves et al.	—	3.8 ± 2.4 (0–10)
Okumura et al.	66.04%	—
Patel et al.	99.16%	3.2 ± 1.7 (0–11)
Puspitasari et al.	—	—
Rodis et al.	—	—
Roth et al.	—	4.2 ± 2.1 (0–11)
Samir Abdin et al.	95.00%	7.2 (1–16)
Santos et al.	65.00%	—
Schwartz et al.	—	2.8 ± 0.9 (1–4)
Tan et al.	79.00%	—
Troncoso-Marino et al.	29.40%	—
Vande Griend et al.	12.14%	—
Yang et al.	98.04%	4.8
Zhang et al.	45.63%	0.88 ± 1.40 (0–8)
Zhao	74.04%	1.4

a DRPs mean per patient = total number of DRPs/sample size.

b Percentage of patients had at least one DRP = the number of patients had at least one DRP/sample size*100%.

Twenty studies reported the types of DRPs: the most common type of DRPs was in the primary domain “treatment safety” (1,138 DRPs, 41.62%), the subdomain of which was “patient suffers, or could suffer, from an adverse drug event” (Gomez et al., 2009; Zhao, 2012; Tan et al., 2014; Mendonca et al., 2016; Rodis et al., 2017; Schwartz et al., 2017; Benson et al., 2018; Yang et al., 2018; Hazen et al., 2019; Khera et al., 2019; Neves et al., 2019; Santos et al., 2019; Zhang et al., 2020a; Zhang et al., 2020b; Chung et al., 2020; Gerard et al., 2020; Samir Abdin et al., 2020); the second was “other” (899 DRPs, 32.88%) (Gomez et al., 2009; Tan et al., 2014; Mendonca et al., 2016; Rodis et al., 2017; Vande Griend et al., 2018; Hazen et al., 2019; Khera et al., 2019; Neves et al., 2019; Santos et al., 2019; Zhang et al., 2020a; Zhang et al., 2020b; Chung et al., 2020; Puspitasari et al., 2020), with the common subdomain “unnecessary drug-treatment” (867 DRPs, 31.71%) (Gomez et al., 2009; Tan et al., 2014; Mendonca et al., 2016; Rodis et al., 2017; Vande Griend et al., 2018; Hazen et al., 2019; Khera et al., 2019; Neves et al., 2019; Santos et al., 2019; Zhang et al., 2020a; Zhang et al., 2020b; Chung et al., 2020; Puspitasari et al., 2020); the last was “treatment effectiveness” (697 DRPs, 25.49%) (Gomez et al., 2009; Nadir et al., 2014; Mendonca et al., 2016; Hazen et al., 2019; Khera et al., 2019; Neves et al., 2019; Santos et al., 2019; Zhang et al., 2020a; Zhang et al., 2020b; Chung et al., 2020), with the common subdomains “no effect of drug treatment despite correct use” (460 DRPs, 16.83%) (Gomez et al., 2009; Nadir et al., 2014; Mendonca et al., 2016; Hazen et al., 2019; Khera et al., 2019; Neves et al., 2019; Santos et al., 2019; Zhang et al., 2020a; Zhang et al., 2020b; Chung et al., 2020) and “effect of drug treatment not optimal” (205 DRPs, 7.50%) (Zhang et al., 2020a; Zhang et al., 2020b). The types of DRPs are detailed in Table 4.

**TABLE 4:** The classifications of types and causes of DRPs of included studies.

\rotate{Study}	\rotate{Benson et al.}	\rotate{Chung et al.}	\rotate{Gerard et al.}	\rotate{Gisev et al.}	\rotate{Gomez et al.}	\rotate{Hazen et al.}	\rotate{Hooper et al.}	\rotate{Khera et al.}	\rotate{Mendonca et al.}	\rotate{Nadir et al.}	\rotate{Neves et al.}	\rotate{Okumura et al.}	\rotate{Puspitasari et al.}	\rotate{Rodis et al.}	\rotate{Samir abdin et al.}	\rotate{Santos et al.}	\rotate{Schwartz et al.}	\rotate{Tan et al.}	\rotate{Troncoso-marinoet al.}	\rotate{Vande griend et al.}	\rotate{Yang et al.}	\rotate{Zhang et al.}	\rotate{Zhao}	\rotate{Total n (%)}
**Types of DRPs, n (%)**																								
**P1**	—	**2**	—	—	**147**	**146**	—	**6**	**16**	**4**	**19**	—	—	—	—	**108**	—	—	—	—	—	**249**	—	**697 (25.49)**
P1.1	—	2	—	—	147	146	—	6	16	4	19	—	—	—	—	108	—	—	—	—	—	12	—	460 (16.83)
P1.2	—	—	—	—	—	—	—	—	—	—	—	—	—	—	—	—	—	—	—	—	—	205	—	205 (7.50)
P1.3	—	—	—	—	—	—	—	—	—	—	—	—	—	—	—	—	—	—	—	—	—	32	—	32 (1.17)
**P2**	**60**	**130**	**91**	—	**70**	**213**	**40**	**9**	**98**	**36**	**40**	—	—	**81**	**26**	**105**	**25**	**23**	—	—	**20**	**42**	**29**	**1,138 (41.62)**
P2.1	60	130	91	—	70	213	40	9	98	36	40	—	—	81	26	105	25	23	—	—	20	42	29	1,138 (41.62)
**P3**	—	**55**	—	—	**13**	**307**	—	**23**	**49**	—	**42**	—	**2**	**79**	—	**238**	—	**15**	—	**5**	—	**71**	—	**899 (32.88)**
P3.1	—	55	—	—	13	307	—	22	49	—	42	—	2	79	—	238	—	15	—	5	—	40	—	867 (31.71)
P3.2	—	—	—	—	—	—	—	1	—	—	—	—	—	—	—	—	—	—	—	—	—	31	—	32 (1.17)
**Causes of DRPs, n (%)**																								
**C1**	**613**	**105**	**563**	**116**	**28**	**735**	**508**	**99**	**65**	**24**	**138**	**59**	**44**	**429**	**170**	**614**	**92**	**103**	**11,717**	**55**	**219**	**143**	**425**	**17,064 (80.38)**
C1.1	70	—	226	—	—	124	252	31	—	3	—	—	16	168	20	—	39	29	10,461	—	8	29	122	11,598 (54.63)
C1.2^a^	340	55	114	—	13	307	19	35	49	11	42	—	27	79	81	238	—	15	—	5	27	15	131	1,603 (7.55)
C1.3	11	—	29	—	—	28	33	—	—	1	—	54	1	1	11	—	40	20	72	1	12	1	107	422 (1.99)
C1.4	—	—	—	—	—	—	146	—	—	5	—	5	—	—	—	—	3	—	1,184	—	10	12	50	1,415 (6.67)
C1.5	192	50	194	—	15	276	58	33	16	4	96	—	—	181	58	376	10	39	—	49	136	56	15	1854 (8.73)
C1.6	—	—	—	—	—	—	—	—	—	—	—	—	—	—	—	—	—	—	—	—	—	30	—	30 (0.14)
**C2**	—	—	—	—	**128**	—	**39**	—	—	**1**	—	**11**	—	—	—	—	—	—	—	—	**4**	**3**	—	**186 (0.88)**
C2.1	—	—	—	—	—	—	39	—	—	1	—	11	—	—	—	—	—	—	—	—	4	3	—	58 (0.27)
**C3**	**304**	**60**	**168**	—	—	**73**	**189**	**25**	**95**	**16**	**120**	**50**	**196**	**308**	**89**	**290**	**12**	**30**	—	**11**	**85**	**57**	**55**	**2,233 (10.52)**
C3.1	84	19	75	—	—	—	—	12	57	13	86	12	54	262	43	175	1	12	—	11	23	25	—	964 (4.54)
C3.2	220	41	93	—	—	—	—	13	38	3	34	22	142	46	46	115	11	18	—	—	12	8	—	862 (4.06)
C3.3	—	—	—	—	—	—	—	—	—	—	—	—	—	—	—	—	—	—	—	—	—	20	—	20 (0.09)
C3.4	—	—	—	—	—	—	—	—	—	—	—	—	—	—	—	—	—	—	—	—	—	4	—	4 (0.02)
**C4**	—	—	—	—	—	—	—	—	—	—	—	—	—	—	—	—	—	—	—	—	—	**9**	—	**9 (0.04)**
C4.1	—	—	—	—	—	—	—	—	—	—	—	—	—	—	—	—	—	—	—	—	—	1	—	1 (0.00)
C4.2	—	—	—	—	—	—	—	—	—	—	—	—	—	—	—	—	—	—	—	—	—	8	—	8 (0.04)
**C5**	—	—	—	**13**	—	—	—	—	—	**40**	—	—	—	—	—	—	**9**	**90**	—	—	**7**	**3**	—	**162 (0.76)**
C5.1	—	—	—	—	—	—	—	—	—	—	—	—	—	—	—	—	6	90	—	—	—	1	—	97 (0.46)
C5.2	—	—	—	13	—	—	—	—	—	40	—	—	—	—	—	—	—	—	—	—	7	—	—	60 (0.28)
C5.3	—	—	—	—	—	—	—	—	—	—	—	—	—	—	—	—	—	—	—	—	—	2	—	2 (0.01)
**C6**	—	—	—	**45**	—	—	**73**	—	—	—	—	**8**	—	**126**	—	—	—	—	—	—	—	**7**	—	**259 (1.22)**
C6.1	—	—	—	—	—	—	—	—	—	—	—	8	—	—	—	—	—	—	—	—	—	5	—	13 (0.06)
C6.3	—	—	—	—	—	—	—	—	—	—	—	—	—	—	—	—	—	—	—	—	—	1	—	1 (0.00)
C6.4	—	—	—	—	—	—	73	—	—	—	—	—	—	—	—	—	—	—	—	—	—	1	—	74 (0.35)
**C7**	**37**	**4**	—	—	—	—	—	**9**	**42**	**54**	**124**	—	—	**205**	**15**	**525**	—	**5**	—	—	**72**	**162**	**64**	**1,318 (6.21)**
C7.1	37	—	—	—	—	—	—	—	—	—	—	—	—	136	—	—	—	—	—	—	24	63	—	260 (1.22)
C7.2	—	—	—	—	—	—	—	—	—	—	—	—	—	69	—	—	—	—	—	—	—	2	—	71 (0.33)
C7.3	—	—	—	—	—	—	—	—	—	—	—	—	—	—	—	—	—	—	—	—	—	9	—	9 (0.04)
C7.4	—	—	—	—	—	—	—	—	—	—	—	—	—	—	—	—	—	—	—	—	—	22	—	22 (0.10)
C7.5	—	—	—	—	—	—	—	—	—	—	—	—	—	—	—	—	—	—	—	—	—	4	—	4 (0.02)
C7.6	—	—	—	—	—	—	—	—	—	—	—	—	—	—	—	—	—	5	—	—	—	1	—	6 (0.03)
C7.7	—	—	—	—	—	—	—	—	—	—	—	—	—	—	—	—	—	—	—	—	—	43	—	43 (0.20)
C7.8	—	—	—	—	—	—	—	—	—	—	—	—	—	—	—	—	—	—	—	—	6	1	—	7 (0.03)
C7.9	—	—	—	—	—	—	—	—	—	—	—	—	—	—	—	—	—	—	—	—	5	17	27	49 (0.23)
**C9**	**126**	—	**38**	**13**	—	**125**	—	—	—	—	—	**9**	—	**326**	—	—	—	—	—	**2**	**103**	**78**	**15**	**835 (3.93)**
C9.1	126	—	—	—	—	125	—	—	—	—	—	9	—	272	—	—	—	—	—	2	73	63	—	670 (3.16)
C9.2	—	—	38	13	—	—	—	—	—	—	—	—	—	54	—	—	—	—	—	—	30	6	15	156 (0.73)
C9.3	—	—	—	—	—	—	—	—	—	—	—	—	—	—	—	—	—	—	—	—	—	9	—	9 (0.04)

The bold values are the classifications of DRPs in primary domain.

a “Unnecessary drug-treatment” also was included in “No indication for drug”; #: C1.1 OR C1.3; †: C3.2 OR C3.4.

Types of DRPs. P1, P1: treatment effectiveness (P1.1: no effect of drug treatment despite correct use, P1.2: effect of drug treatment not optimal, P1.3: untreated symptoms or indication); P2: treatment safety (P2.1: adverse drug event (possibly) occurring); P3: other (P3.1: unnecessary drug treatment, P3.2: unclear problem/complaint). Causes of DRPs. C1: drug selection (C1.1: inappropriate drug according to guidelines/formulary, C1.2: no indication for drug, C1.3: inappropriate combination of drugs, or drugs and herbal medications, or drugs and dietary supplements, C1.4: inappropriate duplication of therapeutic group or active ingredient, C1.5: no or incomplete drug treatment in spite of existing indication, C1.6: too many different drugs/active ingredients prescribed for indication); C2: drug form (C2.1: inappropriate drug form/formulation); C3: dose selection (C3.1: drug dose too low, C3.2: drug dose of a single active ingredient too high, C3.3: dosage regimen not frequent enough, C3.4: dosage regimen too frequent); C4: treatment duration (C4.1: duration of treatment too short, C4.2: duration of treatment too long); C5: dispensing (C5.1: prescribed drug not available, C5.2: Necessary information not provided or incorrect advice provided, C5.3: Wrong drug, strength or dosage advised); C6: drug use process (C6.1: inappropriate timing of administration or dosing intervals by a health professional, C6.3: drug over-administered by a health professional, C6.4: drug not administered at all by a health professional); C7: patient related (C7.1: patient intentionally uses/takes less drug than prescribed or does not take the drug at all for whatever reason, C7.2: patient uses/takes more drug than prescribed, C7.3: patient abuses drug, C7.4: patient decides to use unnecessary drug, C7.5: patient takes food that interacts, C7.6: patient stores drug inappropriately, C7.7: inappropriate timing or dosing intervals, C7.8: patient unintentionally administers/uses the drug in a wrong way, C7.9: patient physically unable to use drug/form as directed); C9: Other (C9.1: no or inappropriate outcome monitoring, C9.2: other causes, C9.3: no obvious cause)

A total of 23 studies reported the causes of DRPs: the most common cause of DRPs was in the primary domain “drug selection” (17,064 DRPs, 80.38%) (Gisev et al., 2009; Gomez et al., 2009; Hooper et al., 2009; Zhao, 2012; Nadir et al., 2014; Tan et al., 2014; Mendonca et al., 2016; Okumura et al., 2016; Rodis et al., 2017; Schwartz et al., 2017; Benson et al., 2018; Vande Griend et al., 2018; Yang et al., 2018; Hazen et al., 2019; Khera et al., 2019; Neves et al., 2019; Santos et al., 2019; Zhang et al., 2020a; Zhang et al., 2020b; Chung et al., 2020; Gerard et al., 2020; Puspitasari et al., 2020; Samir Abdin et al., 2020; Troncoso-Marino et al., 2020), with the common subdomains “inappropriate drug according to guidelines/formulary” (11,598 DRPs, 54.63%) (Hooper et al., 2009; Zhao, 2012; Nadir et al., 2014; Tan et al., 2014; Rodis et al., 2017; Schwartz et al., 2017; Benson et al., 2018; Yang et al., 2018; Hazen et al., 2019; Khera et al., 2019; Zhang et al., 2020a; Zhang et al., 2020b; Gerard et al., 2020; Puspitasari et al., 2020; Samir Abdin et al., 2020; Troncoso-Marino et al., 2020), “no or incomplete drug treatment in spite of existing indication” (1854 DRPs, 8.73%) (Gomez et al., 2009; Hooper et al., 2009; Zhao, 2012; Nadir et al., 2014; Tan et al., 2014; Mendonca et al., 2016; Rodis et al., 2017; Schwartz et al., 2017; Benson et al., 2018; Vande Griend et al., 2018; Yang et al., 2018; Hazen et al., 2019; Khera et al., 2019; Neves et al., 2019; Santos et al., 2019; Zhang et al., 2020a; Zhang et al., 2020b; Chung et al., 2020; Gerard et al., 2020; Samir Abdin et al., 2020), “no indication for drug” (1,603 DRPs, 7.55%) (Gomez et al., 2009; Hooper et al., 2009; Zhao, 2012; Nadir et al., 2014; Tan et al., 2014; Mendonca et al., 2016; Rodis et al., 2017; Benson et al., 2018; Vande Griend et al., 2018; Yang et al., 2018; Hazen et al., 2019; Khera et al., 2019; Neves et al., 2019; Santos et al., 2019; Zhang et al., 2020a; Zhang et al., 2020b; Chung et al., 2020; Gerard et al., 2020; Puspitasari et al., 2020; Samir Abdin et al., 2020), and “too many different drugs/active ingredients prescribed for indication” (1,415 DRPs, 6.67%) (Hooper et al., 2009; Zhao, 2012; Nadir et al., 2014; Okumura et al., 2016; Schwartz et al., 2017; Yang et al., 2018; Zhang et al., 2020a; Zhang et al., 2020b; Troncoso-Marino et al., 2020). Secondly, the cause of 2,233 DPRs was reported in the primary domain “dose selection,” accounting for 10.52% (Hooper et al., 2009; Zhao, 2012; Nadir et al., 2014; Tan et al., 2014; Mendonca et al., 2016; Okumura et al., 2016; Rodis et al., 2017; Schwartz et al., 2017; Benson et al., 2018; Vande Griend et al., 2018; Yang et al., 2018; Hazen et al., 2019; Khera et al., 2019; Neves et al., 2019; Santos et al., 2019; Zhang et al., 2020a; Zhang et al., 2020b; Chung et al., 2020; Gerard et al., 2020; Puspitasari et al., 2020; Samir Abdin et al., 2020), with the common subdomains “drug dose too low” (964 DRPs, 4.54%) (Nadir et al., 2014; Tan et al., 2014; Mendonca et al., 2016; Okumura et al., 2016; Rodis et al., 2017; Schwartz et al., 2017; Benson et al., 2018; Vande Griend et al., 2018; Yang et al., 2018; Khera et al., 2019; Neves et al., 2019; Santos et al., 2019; Zhang et al., 2020a; Zhang et al., 2020b; Chung et al., 2020; Gerard et al., 2020; Puspitasari et al., 2020; Samir Abdin et al., 2020) and “drug dose too high” (862 DRPs, 4.06%) (Nadir et al., 2014; Tan et al., 2014; Mendonca et al., 2016; Okumura et al., 2016; Rodis et al., 2017; Schwartz et al., 2017; Benson et al., 2018; Yang et al., 2018; Khera et al., 2019; Neves et al., 2019; Santos et al., 2019; Zhang et al., 2020a; Zhang et al., 2020b; Chung et al., 2020; Gerard et al., 2020; Puspitasari et al., 2020; Samir Abdin et al., 2020). Thirdly, the cause of 1,318 DRPs (6.21%) was reported in “patient related” (Zhao, 2012; Nadir et al., 2014; Tan et al., 2014; Mendonca et al., 2016; Rodis et al., 2017; Benson et al., 2018; Yang et al., 2018; Khera et al., 2019; Neves et al., 2019; Santos et al., 2019; Zhang et al., 2020a; Zhang et al., 2020b; Chung et al., 2020; Samir Abdin et al., 2020), with the common subdomain “patient intentionally uses/takes less drug than prescribed or does not take the drug at all for whatever reason” (260 DRPs, 1.22%) (Rodis et al., 2017; Benson et al., 2018; Yang et al., 2018; Zhang et al., 2020a; Zhang et al., 2020b) and other unspecified causes for poor medication adherence. In addition, the cause of 835 DRPs (3.93%) was reported in the primary domain “other” (Gisev et al., 2009; Zhao, 2012; Okumura et al., 2016; Rodis et al., 2017; Benson et al., 2018; Vande Griend et al., 2018; Yang et al., 2018; Hazen et al., 2019; Zhang et al., 2020a; Zhang et al., 2020b; Gerard et al., 2020), with the common subdomain “no or inappropriate outcome monitoring” (670 DRPs, 3.16%) (Okumura et al., 2016; Rodis et al., 2017; Benson et al., 2018; Vande Griend et al., 2018; Yang et al., 2018; Hazen et al., 2019; Zhang et al., 2020a; Zhang et al., 2020b). The causes of DRPs are detailed in Table 4.

### Risk Factors for DRPs

Eleven studies reported risk factors of DRPs (Patel et al., 2005; Gomez et al., 2009; Nadir et al., 2014; Mendonca et al., 2016; Vande Griend et al., 2018; Yang et al., 2018; Khera et al., 2019; Neves et al., 2019; Santos et al., 2019; Zhang et al., 2020a; Zhang et al., 2020b; Troncoso-Marino et al., 2020). Of these, seven studies reported that the number of medicines taken at the same time was a positive factor for the occurrence of DRPs (Nadir et al., 2014; Mendonca et al., 2016; Vande Griend et al., 2018; Yang et al., 2018; Santos et al., 2019; Zhang et al., 2020a; Zhang et al., 2020b; Troncoso-Marino et al., 2020). Four studies reported that age was a positive factor for the occurrence of DRPs (Nadir et al., 2014; Vande Griend et al., 2018; Yang et al., 2018; Zhang et al., 2020a; Zhang et al., 2020b). However, 1 study reported no statistically significant difference in the occurrence of DRPs between the age group of over 65 years and the age group of under 65 years (Gomez et al., 2009). Two studies showed that the number of comorbidities was a positive factor for the occurrence of DRPs (Santos et al., 2019; Zhang et al., 2020a; Zhang et al., 2020b). Moreover, factors including the number of visits (Troncoso-Marino et al., 2020), clinical pharmacy priority score (Vande Griend et al., 2018), quality of life score EQ-5D (Zhang et al., 2020a; Zhang et al., 2020b), and frailty level (Khera et al., 2019), which reflected a patient’s health status, were also positively associated with the occurrence of DRPs. Moreover, two studies mentioned that creatinine clearance rate (Patel et al., 2005) and hypertension (Neves et al., 2019) were related to the occurrence of DRPs, respectively. Three studies reported the effect of gender on the occurrence of DRP: one study (Troncoso-Marino et al., 2020) showed that women had a higher risk of DRPs than men with an OR ranging from 1.12 (95% CI: 1.10–1.14) to 1.24 (95% CI: 1.19–1.30); however, the other two studies (Gomez et al., 2009; Nadir et al., 2014) showed no statistical difference in the effect of gender on the occurrence of DRPs. One study (Zhang et al., 2020a; Zhang et al., 2020b) reported that patients with poorer medication adherence were more likely to have DRPs. In addition, six studies (Gomez et al., 2009; Zhao, 2012; Okumura et al., 2016; Schwartz et al., 2017; Hazen et al., 2019; Neves et al., 2019) reported a strong association between certain drug categories and the occurrence and frequency of DRPs, but it might be because that some populations themselves take more drugs of certain categories.

### Intervenability of DRPs

Interventions were at the prescriber, patient, drug, and other levels. Interventions at the prescriber level mainly included “intervention proposed to prescriber” (Zhang et al., 2020a; Zhang et al., 2020b). Interventions at the patient level mainly included “patient referred to prescriber” (Hooper et al., 2009; Tan et al., 2014; Zhang et al., 2020a; Zhang et al., 2020b). Moreover, interventions at the drug level mainly included “drug changed to …,” “dosage changed to …,” “drug paused or stopped,” “drug started,” and “instructions for (the time of using drugs) changed to …” (Tan et al., 2014; Mendonca et al., 2016; Okumura et al., 2016; Schwartz et al., 2017; Vande Griend et al., 2018; Yang et al., 2018; Hazen et al., 2019; Khera et al., 2019). Other interventions included drug monitoring (Hooper et al., 2009; Schwartz et al., 2017; Yang et al., 2018; Khera et al., 2019). Twelve studies reported the rates of interventions accepted by prescribers (the rates were 40.9% (Patel et al., 2005), 49.8% (Chung et al., 2020), 67.9% (Neves et al., 2019), 68.1% (Yang et al., 2018), 70% (Hooper et al., 2009), 76% (Gisev et al., 2009), 81.6% (Zhang et al., 2020a; Zhang et al., 2020b), 87.7% (Samir Abdin et al., 2020), 89% (Okumura et al., 2016), 90.2% (Gomez et al., 2009), 90.9% (Schwartz et al., 2017), and 94% (Roth et al., 2013), respectively), with a median (IQR) of 78.8% (22.3%). Four studies reported the rates of implemented interventions (the rates were 42.8% (Chung et al., 2020), 60.9% (Yang et al., 2018), 67.40% (Zhang et al., 2020a; Zhang et al., 2020b), and 70% (Benson et al., 2018), respectively), with a median (IQR) of 64.15% (16.85%). Four studies reported the rates of solved DRPs (the rates were 42.6% (Santos et al., 2019), 73.5% (Tan et al., 2014), 80.47% (Hazen et al., 2019), and 87.8% (Gomez et al., 2009), respectively), with a median (IQR) of 76.99% (26.09%).

## Discussion

### Results of This Research

This systematic review included current studies related to DRPs of patients in primary health care institutions and provided the epidemiological characteristics of DRPs. The results showed that the incidence of DRPs of patients in primary health care institutions was serious, with a median (IQR) of 70.04% (59%) for the percentage of patients with at least one DRP and a median (IQR) of 3.4 (2.8) for the average number of DRPs per patient, suggesting that attention should be paid to the DRPs of this population. Similar results were found in another systematic review of the home-dwelling older adults, which showed that the average number of DRPs per patient was 4.16 (1.37–10) (Plácido et al., 2020). “Therapeutic safety” accounted for the largest proportion of the types of DRPs, i.e., “patient suffers, or could suffer, from an adverse drug event.” Moreover, the causes of DRPs were mainly in the prescribing process, including drug and dose selection, suggesting that continuing education and training for prescribers in primary health care institutions should be emphasized. Moreover, poor medication adherence in the use process was also an important cause of DRPs, suggesting that attention should also be paid to improving the adherence of patients. Meanwhile, it also showed that medication reviews should be conducted throughout the whole medication process with the patients' involvement. Identifying actual or potential risk factors of DRPs should be considered an important part of safe medication. The result of this research suggested that the number of medicines, age, and disease condition might be risk factors for the occurrence of DRPs, but most of the included studies involved middle-aged and elderly patients as study subjects, which might result in an incomplete summary of risk factors. DRPs are preventable (Pereira et al., 2012), and clinical pharmacists can use DRP risk assessment tools that have been developed (Puumalainen et al., 2020) to identify risk factors of DRPs. Pharmacists could carry out interventions at the prescriber, patient, drug, and other levels. The results of this research showed that the median (IQR) for the rates of accepted interventions was 78.8% (22.3%), the median (IQR) for the rates of implemented interventions was 64.15% (16.85%), and the median (IQR) for the rates of solved DRPs was 76.99% (26.09%), suggesting the intervention ability of DRPs and also reflecting the important role of pharmacists in DRPs intervention. The interventions of DRPs have been conducted in a number of studies, and serious consequences of DRPs have been avoided through pharmacists' intervention (Freyer et al., 2018; Ylä-Rautio et al., 2020).

### Innovation of This Research

To the best of our knowledge, this research is the first to systematically review the characteristics of DRPs in primary health care institutions, focusing on this population with poorer medical care and more serious condition of DRPs and providing evidence-based medical evidence for subsequent researches about the interventions of DRPs. Therefore, this research is innovative in the content, and at the same time, this systematic review strictly adhered to the methodology of evidence-based medicine.

### Limitations of This Research

The research aimed to explore the overall characteristics of DRPs in primary care institutions, so there was heterogeneity among the included studies in terms of study design, age, disease condition, and level of medical care, which might have some impacts on the results. In addition, some studies did not report the DRPs classification systems used or the systems were different in several studies, so the types and causes of DRPs were reclassified using PCNE V 9.1 in this research. However, this might result in inappropriate classifications due to incomplete information. More importantly, the quality and comprehensiveness of source data had some limitations: there was only one high-quality study and the rest were of moderate quality; the common English and Chinese databases, search engines, and the reference lists of included studies were searched, but there might still be inevitable omissions of qualified studies. Despite these limitations, this research provided a comprehensive and systematic review of the current studies related to DRPs of patients in primary care institutions.

### Future Research Directions

Firstly, this research found that current studies related to DRPs in primary care institutions mostly focused on elderly patients, with less attention paid to the children. However, DRPs such as unlicensed and off-label prescriptions were also common among children in primary care institutions (Andrade et al., 2020; Kaushal et al., 2001; Glanz et al., 2016), especially for children with chronic diseases (Quittner et al., 2008; Walsh et al., 2011). Therefore, future studies could research the characteristics of DRPs in this population. Secondly, we found that the causes of DRPs were mainly in the prescribing process, which might be due to poor medical care in primary health institutions. So, in addition to continuing education and training for prescribers, future studies could explore whether it will be possible to improve the level of prescribing with the help of internet technology in primary care institutions (Mcmahon et al., 2005; Avery et al., 2012; Oldenburg et al., 2015), to achieve regional medical homogenization. Thirdly, from the included studies in this systematic review, we found that the scope of medication reviews in certain studies was limited to prescription drugs, without over-the-counter drugs and health supplements. Fourthly, some patients did not get involved in medication reviews, which might lead pharmacists to ignore DRPs in the medication use process, such as poor medication adherence and lack of medication knowledge. In the future, comprehensive medication reviews with the involvement of patients should be conducted in researches and practice (Kovačević et al., 2017; Kari et al., 2018). Finally, future researches should pay more attention to quality control and refer to the items of quality assessment scales and report specification files. For example, researchers of cross-sectional studies should clarify the source of data, inclusion and exclusion criteria, and the time to identify patients. At the same time, sufficient and appropriate sample sizes should be ensured through statistical computing.

## Conclusion

In this research, we systematically reviewed the existing studies on DRPs of patients in primary care institutions and found that the incidence of DRPs and the average number of DRPs per patient in primary care institutions were high, mainly in the prescription process. Risk factors such as the number of medicines, age, and disease condition were positively associated with the occurrence of DRPs, and this population with those risk factors should be closely monitored. In addition, the results showed that pharmacists might play an important role in the identification and intervention of DRPs.

## Data Availability

The original contributions presented in the study are included in the article/Supplementary Material; further inquiries can be directed to the corresponding author.

## Abbreviations

DRP, drug-related problem; PCNE, Pharmaceutical Care Network Europe; IQR, inter-quartile range.
