# Supplementary material for: Drug-Related Problems of Patients in Primary Health Care Institutions: A Systematic Review
Source: Front Pharmacol. 2021 Aug 17;12:698907. doi: 10.3389/fphar.2021.698907 (PMC8418140; doi:10.3389/fphar.2021.698907)
Supplement: Supplementary file 1 [file DataSheet1.docx]

**Appendix 1 Search strategies**

1. **English databases**

**1.1 PubMed**

(("Primary Health Care"[MeSH Terms]) OR primary health care[Title/Abstract] OR (primary medical care[Title/Abstract] OR primary care[Title/Abstract] OR Primary Healthcare[Title/Abstract] OR community[Title/Abstract] OR communities[Title/Abstract] OR town[Title/Abstract] OR towns[Title/Abstract] OR township[Title/Abstract] OR townships[Title/Abstract] OR county[Title/Abstract] OR counties[Title/Abstract] OR village[Title/Abstract] OR villages[Title/Abstract] OR rural[Title/Abstract] OR ("[Rural Health Services](https://www.ncbi.nlm.nih.gov/mesh/68019035)"[MeSH Terms]) OR clinic[Title/Abstract] OR clinics[Title/Abstract])) AND (drug related problem*[Title/Abstract] OR medication related problem*[Title/Abstract] OR medicine related problem*[Title/Abstract] OR drug therapy problem*[Title/Abstract] OR medication therapy problem*[Title/Abstract] OR medicine therapy problem*[Title/Abstract] OR therapy related problem*[Title/Abstract])

**1.2 Cochrane library**

#1 MeSH descriptor: [Primary Health Care] explode all tree

#2 (Primary Health Care):ti.ab.kw

#3 (primary medical care):ti.ab.kw

#4 (primary care):ti.ab.kw

#5 (Primary Healthcare):ti.ab.kw

#6 (community):ti.ab.kw

#7 (communities):ti.ab.kw

#8 (town):ti.ab.kw

#9 (towns):ti.ab.kw

#10 (township):ti.ab.kw

#11 (townships):ti.ab.kw

#12 (county):ti.ab.kw

#13 (counties):ti.ab.kw

#14 (village):ti.ab.kw

#15 (villages):ti.ab.kw

#16 (rural):ti.ab.kw

#17 MeSH descriptor: [[Rural Health Services](https://www.ncbi.nlm.nih.gov/mesh/68019035)] explode all tree

#18 (clinic):ti.ab.kw

#19 (clinics):ti.ab.kw

#20 #1 OR #2 OR #3 OR #4 OR #5 OR #6 OR #7 OR #8 OR #9 OR #10 OR #11 OR #12 OR #13 OR #14 OR #15 OR #16 OR #17 OR #18 OR #19

#21 (“drug related problem*”):ti.ab.kw

#22 (“medication related problem*”):ti.ab.kw

#23 (“medicine related problem*”):ti.ab.kw

#24 (“drug therapy problem*”):ti.ab.kw

#25 (“medication therapy problem*”):ti.ab.kw

#26 (“medicine therapy problem*”):ti.ab.kw

#27 (“therapy related problem*”):ti.ab.kw

#28 #21 OR #22 OR #23 OR #24 OR #25 OR #26 OR #27

#29 #20 AND #28

**1.3 EMbase**

#1 primary medical care.mp. or exp primary medical care/

#2 primary health care.mp. or exp primary health care/

#3 primary care.mp.

#4 Primary Healthcare.mp.

#5 community.mp. or exp community/

#6 communities.mp.

#7 town.mp.

#8 towns.mp.

#9 township.mp.

#10 townships.mp.

#11 county.mp.

#12 counties.mp.

#13 village.mp.

#14 villages.mp.

#15 rural.mp. or exp rural hospital/ or exp rural health care/

#16 clinic.mp.

#17 clinics.mp.

#18 drug related problem*.mp.

#19 medication related problem*.mp.

#20 medicine related problem*.mp.

#21 drug therapy problem*.mp.

#22 medication therapy problem*.mp.

#23 medicine therapy problem*.mp.

#24 therapy related problem*.mp.

#25 1 or 2 or 3 or 4 or 5 or 6 or 7 or 8 or 9 or 10 or 11 or 12 or 13 or 14 or 15 or 16 or 17

#26 18 or 19 or 20 or 21 or 22 or 23 or 24

#27 25 and 26

1. **Chinese databases**
   1. **CNKI**

高级检索：

主题：用药相关问题 + 药物相关问题 + 药物治疗相关问题 + 药物治疗问题 + 治疗相关问题

主题：基层 + 社区 + 街道 + 乡镇 + 农村 + 卫生服务中心 + 卫生院 + 卫生室 + 门诊部 + 诊所

- 1. **CBM**

("初级卫生保健"[不加权:扩展] OR "初级卫生保健"[常用字段:智能] OR "乡村卫生服务"[不加权:扩展] OR "乡村卫生服务"[常用字段:智能] OR "基层"[常用字段:智能] OR "社区"[常用字段:智能] OR "街道"[常用字段:智能] OR "乡镇"[常用字段:智能] OR "农村 OR"[常用字段:智能] OR "卫生服务中心"[常用字段:智能] OR "卫生院"[常用字段:智能] OR "卫生室"[常用字段:智能] OR "门诊部"[常用字段:智能] OR "诊所"[常用字段:智能]) AND [("用药相关问题"[常用字段:智能] OR "药物相关问题"[常用字段:智能] OR "药物治疗相关问题"[常用字段:智能] OR "药物治疗问题"[常用字段:智能] OR "治疗相关问题"[常用字段:智能])](http://libdb.csu.edu.cn/rwt/ZGSWYX/http/P75YPLUUNFYG85LFMRYGCZ3PMNYDVPBR/zh/javascript:toDoRelimitSearch();)

- 1. **VIP**

(M=用药相关问题 OR R=用药相关问题 OR M=药物相关问题 OR R=药物相关问题 OR M=药物治疗相关问题 OR R=药物治疗相关问题 OR M=药物治疗问题 OR R=药物治疗问题 OR M=治疗相关问题 OR R=治疗相关问题) AND (M=基层 OR R=基层 OR M=社区 OR R=社区 OR M=街道 OR R=街道 OR M=乡镇 OR R=乡镇 OR M=农村 OR R=农村 OR M=卫生服务中心 OR R=卫生服务中心 OR M=卫生院 OR R=卫生院 OR M=卫生室 OR R=卫生室 OR M=门诊部 OR R=门诊部 OR M=诊所 OR R=诊所)

- 1. **WanFang**

专业检索：

(主题:("用药相关问题") or 主题:("药物相关问题") or 主题:("药物治疗相关问题" or 主题:("药物治疗问题") or 主题:("治疗相关问题")) and (主题:("基层") or 主题:("社区") or 主题:("街道") or 主题:("乡镇") or 主题:("农村") or 主题:("卫生服务中心") or 主题:("卫生院") or 主题:("卫生室") or 主题:("门诊部") or 主题:("诊所"))
